# Facial Recognition of Happiness Is Impaired in Musicians with High Music Performance Anxiety

**DOI:** 10.3389/fpsyt.2018.00005

**Published:** 2018-01-25

**Authors:** Alini Daniéli Viana Sabino, Cristielli M. Camargo, Marcos Hortes N. Chagas, Flávia L. Osório

**Affiliations:** ^1^Department of Neuroscience and Behavior, Ribeirão Preto Medical School, University of São Paulo, Ribeirão Preto, Brazil; ^2^Center for Biological and Health Sciences, Federal University of São Carlos, São Carlos, Brazil; ^3^National Institute for Science and Technology – Translational Medicine (INCT-TM, CNPq), Brasília, Brazil

**Keywords:** music performance anxiety, social cognition, faces, social anxiety, cognitive factors

## Abstract

**Introduction:**

Music performance anxiety (MPA) can be defined as a lasting and intense apprehension connected with musical performance in public. Studies suggest that MPA can be regarded as a subtype of social anxiety. Since individuals with social anxiety have deficits in the recognition of facial emotion, we hypothesized that musicians with high levels of MPA would share similar impairments.

**Objective:**

The aim of this study was to compare parameters of facial emotion recognition (FER) between musicians with high and low MPA.

**Methods:**

150 amateur and professional musicians with different musical backgrounds were assessed in respect to their level of MPA and completed a dynamic FER task. The outcomes investigated were accuracy, response time, emotional intensity, and response bias.

**Results:**

Musicians with high MPA were less accurate in the recognition of happiness (*p* = 0.04; *d* = 0.34), had increased response bias toward fear (*p* = 0.03), and increased response time to facial emotions as a whole (*p* = 0.02; *d* = 0.39).

**Conclusion:**

Musicians with high MPA displayed FER deficits that were independent of general anxiety levels and possibly of general cognitive capacity. These deficits may favor the maintenance and exacerbation of experiences of anxiety during public performance, since cues of approval, satisfaction, and encouragement are not adequately recognized.

## Introduction

Music performance anxiety (MPA) is defined as persistent, intense, and distressing apprehension in situations involving music performance in public. MPA occurs along a continuum of severity and, in its extreme form, affects musical aptitude through physical, behavioral, and cognitive manifestations (1, 2). The experience of this state of anxiety may impair musical performance, leading a significant number of professional musicians to quit their careers (3).

The prevalence of MPA is still uncertain, with rates ranging between 15 and 25% (4). In Brazil, a recent study found that MPA affected 24% of a sample formed by amateur and professional musicians (5).

There is strong evidence for the existence of a genetic component in the etiology of MPA. Studies have described gene-environment interactions, highlighting the associations between genes and a childhood history of social inhibition or shyness and gradual or insidious embarrassing experiences (6). In addition to these factors, cognitive aspects also play a relevant role in MPA. Facial emotion recognition (FER), one of the components of social cognition (7, 8), stands out among these aspects as a key element for social behavior and interaction (9).

There is a substantial amount of evidence from investigations on the relationship between anxiety disorders and FER showing that individuals suffering from anxiety disorders tend to have an enhanced recognition of threatening or disapproving faces and of negative facial emotions (10–13). FER deficits are suggestive of distortions in information processing that may be specific to and associated with the onset of such disorders (13–15). Specifically in respect to MPA, previous studies have shown that affected musicians had more negative cognitions and more catastrophic thoughts when compared with musicians without MPA (16–18). To the best of our knowledge, however, no study to date has dealt with FER in this population. Our hypothesis was that musicians with high MPA would have specific FER deficits (negative bias) that could be associated with the onset and maintenance of the condition.

### Aims of the Study

To compare the performance of musicians with low and high MPA in an FER task based on recognition accuracy, response time, emotional intensity, and response bias.

## Materials and Methods

The study involved a convenience sample of 150 musicians, with 100 (66.7%) “popular” musicians (with no academic background in music) and 50 (33.3%) classical musicians (with an academic background or degree in music) from different regions of the state of São Paulo, Brazil. Popular musicians were recruited at rehearsal sessions of different musical groups, including choirs of catholic and protestant churches and independent bands. The most frequent music genres played by these groups were gospel, country, Brazilian pop (*MPB*), pop rock, samba, and *pagode* (a style connected to samba). Classical/orchestra musicians were recruited at music colleges and conservatories.

We invited musicians who were 18 years old or older, who took part in public performances, and who agreed to participate by providing their signed informed consent. Volunteers who did not complete the assessment instruments and/or the FER task were excluded from the study.

After an initial assessment, the musicians were allocated to two groups according to MPA level (high MPA and low MPA).

### Instruments

The Kenny Music Performance Anxiety Inventory (K-MPAI), translated and adapted to Brazil (19), was used to assess symptoms of anxiety and distress and physiological and memory alterations associated with MPA. The instrument consists of 26 items rated in a *Likert* scale ranging from “strongly disagree” (−3) to “strongly agree” (+3), with higher scores indicating higher levels of MPA. The criteria for inclusion in the group with high MPA were a score ≥−15, established according to the psychometric study by Barbar et al. (20).

The Beck Anxiety Inventory, created by Beck and Steer (21) and translated and adapted to Brazil by Cunha (22), is a self-rating instrument used to assess general aspects of anxiety. It comprises 21 multiple-choice items rated in a five-point *Likert* scale and higher scores indicate higher anxiety levels. In this study, we used the cutoff score of 20 points as an indicator of the presence of anxiety disorders, as suggested by Cunha (22).

To screen for the presence of depressive symptoms in the sample, we used the nine-item version of the Patient Health Questionnaire developed by Kroenke et al. (23) and adapted to Brazil by Osório et al. (24) The items of the questionnaire are rated in a *Likert* scale between 0 (“Never”) and 3 (“Almost everyday”) and refer to symptoms occurring over the 15 days preceding the assessment. The cutoff score of 10 proposed by Osório et al. (24) was used to establish the presence of indicators of depression.

Finally, to assess FER, we used a task called *Touch the Face*, which is a dynamic computerized task consisting in the presentation of 24 short videos with 1.000 frames each and a duration of 10 s. Each video starts with a neutral face (0% emotion) and advances to show the full expression (100%) of basic facial emotions (happiness, sadness, disgust, fear, anger, and surprise). Participants are asked to touch the screen when they recognize the emotion displayed. Each of the six emotions is presented four times in a random order and is portrayed by two actors and two actresses. The task uses stimuli created by Ekman and Friesen (25) and procedures proposed by Arrais et al. (13) (see Supplementary Material for further details).

The study was approved by the local ethics committee (CAAE: 39523314.0.0000.5440), and all volunteers signed the informed consent form before participation.

### Statistical Analysis

The data were organized in a database and analyzed with the Statistical Package for the Social Sciences, version 20.0 (IBM). We used descriptive statistics (mean, SD, and frequency) to characterize the sample. Univariate analyses (ANOVA, chi-square) were used to compare the groups in terms of their clinical and sociodemographic characteristics and the outcome variables of the FER task (accuracy, response time, and emotion intensity). Covariance analyses (ANCOVA) were used to assess the possible effects of confounding variables (years of education, age, anxiety, and depression severity). In addition, a test for the comparison of two proportions was used to assess response biases. To assess the effect size of differences, we used Cohen’s *d*, which was interpreted according to the following classification: small: *d* = 0.2; medium: *d* = 0.5; and large: *d* = 0.8 (26). The level of statistical significance in all tests was *p* < 0.05.

## Results

The musicians were assigned to the high MPA and low MPA groups according to their scores in the K-MPAI, with 74 (49.33%) volunteers presenting indicators of MPA according to the instrument. The sociodemographic and clinical data of the sample are described in Table 1.

**Table 1:** Clinical and demographic data stratified by group.

	Musicians with low MPA, *n*(%)	Musicians with high MPA, *n*(%)	*p*
76 (50.67%)	74 (49.33%)	
**Sex**			
Female	25 (32.9)	25 (33.8)	0.908
Male	51 (67.1)	49 (62.2)	
**Education (years)**			
≤12	25 (32.9)	13 (17.6)	0.031
>12	51 (67.1)	61 (82.4)	
**Background**			
Popular	47 (61.8)	44 (59.4)	0.765
Classic	29 (38.2)	33 (44.6)	
	**Mean (SD)**		
Age	29.7 (±11.97)	24.5 (±7.55)	0.002
BAI score	8.4 (±6.11)	13.7 (±9.30)	<0.001
PHQ-9 score	4.8 (±3.59)	8.5 (±5.66)	<0.001

*MPA, music performance anxiety; BAI, Beck Anxiety Inventory; n, number of subjects in each group; PHQ-9, 9-item Patient Health Questionnaire*.

As shown in Table 1, the group of musicians with high MPA was formed by younger people with more years of education. These musicians also had higher levels of symptoms of depression and anxiety.

In the FER task, the groups were compared in terms of recognition accuracy, response time, emotional intensity, and response bias. Table 2 presents the accuracy data for the two groups.

**Table 2:** Mean and percentage of correct responses (accuracy) in the facial emotion recognition task of musicians with low and high MPA.

Emotion	Musicians with low MPA	Musicians with high MPA	*p*^a^	*d*
**Happiness**				
$\bar{X}(\text{SD})$	3.67 (0.57)	3.43 (0.81)	0.04*	−0.34 (−0.66 to −0.22)
%	92.00	85.00		
**Sadness**				
$\bar{X}(\text{SD})$	2.90 (1.10)	2.66 (1.03)	0.16	−0.22 (−0.54 to −0.10)
%	72.50	69.00		
**Fear**				
$\bar{X}(\text{SD})$	2.23 (1.17)	1.94 (1.22)	0.14	−0.24 (−0.56 to 0.08)
%	55.75	48.50		
**Disgust**				
$\bar{X}(\text{SD})$	2.72 (1.10)	2.55 (1.04)	0.33	0.16 (−0.48 to 0.16)
%	68.00	63.75		
**Anger**				
$\bar{X}(\text{SD})$	2.48 (0.91)	2.27 (1.01)	0.17	−0.21 (−0.54 to 0.10)
%	62.00	56.75		
**Surprise**				
$\bar{X}(\text{SD})$	3.11 (0.93)	2.81 (1.10)	0.07	−0.30 (−0.61 to −0.03)
%	77.75	70.25		
**Total**				
$\bar{X}(\text{SD})$	17.14 (3.47)	15.67 (3.96)	0.02*	−0.40 (−0.71 to −0.07)
%	71.42	65.30		

*d, Cohen’s d (effect size, confidence interval of 95%); MPA, music performance anxiety*; $\bar{X}$, *mean; %, percentage of correct responses*.

*Statistically significant difference between groups.

*^a^ANOVA*.

Table 2 shows that musicians with high MPA had lower FER accuracy in general and specifically for the recognition of happiness, with a medium effect size. The differences in the total score (*F*_1,149_ = 7.93; *p* = 0.006) and for the recognition of happiness (*F*_1,149_ = 8.94, *p* = 0.003) persisted after controlling for age, education, and level of depression and anxiety symptoms. According to the response bias analysis, the group with high MPA misattributed fear to faces more frequently than musicians with low MPA (*p* = 0.03).

The data on the response time for FER are presented in Table 3.

**Table 3:** Mean response time (in seconds) in the facial emotion recognition task for musicians with high and low MPA.

Emotion	Musicians with low APM	Musicians with high APM	*p*^a^	*d*
**Happiness**				
$\bar{X}(\text{SD})$	8.17 (3.29)	8.71 (3.58)	0.33	0.16 (−0.16 to 0.48)
**Sadness**				
$\bar{X}(\text{SD})$	10.57 (4.34)	12.13 (5.08)	0.04*	0.33 (0.01 to 0.65)
**Fear**				
$\bar{X}(\text{SD})$	9.58 (3.77)	10.63 (4.69)	0.13	0.25 (−0.07 to 0.57)
**Disgust**				
$\bar{X}(\text{SD})$	9.56 (3.56)	10.48 (5.22)	0.20	0.21 (−0.11 to 0.53)
**Anger**				
$\bar{X}(\text{SD})$	10.95 (4.44)	11.96 (5.46)	0.21	0.20 (−0.12 to 0.52)
**Surprise**				
$\bar{X}(\text{SD})$	9.63 (4.31)	12.53 (8.06)	0.007*	0.45 (0.13 to 0.77)
**Total**				
$\bar{X}(\text{SD})$	9.83 (3.69)	11.44 (4.42)	0.02*	0.39 (0.07 to 0.72)

*MPA, music performance anxiety; d, Cohen’s d (effect size, confidence interval of 95%); n, number of subjects in each group*; $\bar{X}$, *mean*.

*Statistically significant difference between groups.

*^a^ANOVA*.

The data show that musicians with high MPA took longer to recognize facial emotions compared with musicians with low MPA, as expressed by the total time and by the time to recognize sadness and surprise (medium effect size). After adjusting for age, education, and symptoms of depression and anxiety, significant differences persisted in total time (*F*_1,149_ = 4.53; *p* = 0.035) and time for the recognition of surprise (*F*_1,149_ = 3.97; *p* = 0.05).

In respect to the emotional intensity required for recognition, there were no statistically significant differences between musicians with high and low MPA. However, there was a tendency for volunteers with high MPA to require greater emotional intensity for the recognition of emotions in general (*p* = 0.06) and of sadness (*p* = 0.11) and surprise (*p* = 0.08).

## Discussion

This was a preliminary investigation about the recognition of facial emotions by musicians according to their level of MPA. Our main finding was a deficit in the recognition of happiness in musicians with high MPA compared with those with low MPA, which had a medium effect size.

As mentioned earlier, MPA is characterized by persistent and intense apprehension associated with music performance in public, regardless of the musician’s aptitude, training, or preparation (6, 27), and considered by some authors to be a subtype of social anxiety disorder (6, 28, 29). In addition to earlier reports showing that social anxiety disorder is an important predictor of MPA (5), there is also evidence of significant similarities between the cognitive processing of individuals with social anxiety disorder and musicians with MPA, which supports the view that the theoretical cognitive-behavioral model proposed for social anxiety disorder (30) is also valid for MPA (6).

Therefore, the understanding of this specific condition must depart from previous and consolidated knowledge about social anxiety. In this sense, information processing disturbances at the cognitive, attentional, and memory (30, 31) levels play an important role and favor the maintenance and exacerbation of anxiety symptoms. In respect to FER, the available literature describes alterations in subjects with social anxiety disorder expressed as hypersensitivity, hypervigilance, and negative biases (increased attribution of negative emotions to neutral faces and enhanced recognition of angry/threatening expressions) (10, 32, 33). Consonant with this, musicians with high MPA in our study presented deficits in FER with the predominance of a negative bias, independently of depression and anxiety levels. Here, however, the impairment was in the recognition of signs of social approval, considering the decreased accuracy in the recognition of facial expressions of happiness.

This finding is consonant with the results of previous studies involving subjects with different levels of social anxiety, which showed a decreased capacity to recognize positive feedback in this population (34, 35). This impairment has also been observed in other dimensions of emotional processing (36–38). To many authors, positive emotions are the most easily recognizable because of the tendency of human beings to create positively biased hypotheses about reality and other individuals (39). In general, healthy individuals see others as sources of reward and expect positive social interactions (36). However, according to Clark and Wells (31) and Rapee and Heimberg (30), the others are seen as sources of criticism and threat in social anxiety, which favors the engagement in a negatively biased anticipatory process that precedes the social situation that is feared.

This interpretation bias thwarts the access to and retrieval of existing positive information about other individuals and the environment that could be applied to the new situation at issue. The judgment of reality and individuals is then based on the negative bias of criticism, high expectations, and disapproval (31). Thus occurs the activation of emotionally unpleasant information and, as a result, the disadvantage for the recognition of positive emotions, since the signs of acceptance and reward become inconsistent with the emotions and intentions expected from others (35). In other words, the activation of dysfunctional interpersonal beliefs proper to MPA make positive information difficult to be retrieved and applied in the interpretation of facial expressions, especially during the anxiogenic situation of public performance. Along this line of thought, it should be noted that happy faces were more frequently interpreted as displaying fear by musicians with high MPA.

Another possible explanation for the findings of this study is based on the view proposed by Campbell et al. (37) and Roelofs et al. (40) that the perception of positive feedback in subjects with social anxiety amplifies the desire for social approval and underlying feelings of anxiety, since a particular interpretation appears according to which greater expectations from the audience (manifested through approving expressions like happy faces in this specific case) would mean lower chances of fulfilling them. According to this view, musicians with high MPA would avoid social feedbacks, which could be associated with the deficits in FER described here.

Differently from Leppanen and Hietanen (41), we found no increase in the processing time for facial displays of happiness. For those authors, the increase in the processing time of the positive emotion lends further support to the influence of negative emotional representations that would lead to a delayed and inhibited cognitive appraisal of positive social clues.

The findings described here point to the presence of impaired cognitive functioning in musicians with high MPA that affects emotional recognition and that is independent of general anxiety levels, and possibly of general cognitive capacity as well. This impairment could favor the maintenance and exacerbation of experiences of anxiety during public performance, given that signs of approval, satisfaction, and encouragement are not adequately recognized.

Considering that FER is an important tool in the context of interpersonal interactions, treatments tailored for this population should take into account these specific deficits and involve techniques and pharmacological substances with efficacy at this level.

One limitation of our study was the fact that the participants were not assessed in respect to the presence and level of social anxiety. Future investigations should take this variable into account and include comparison groups with social anxiety to check whether FER parameters are similar to the ones described here. Furthermore, considering that FER is only one of the many domains of social cognition, the investigation of the performance of musicians with high MPA in other tests of social cognition is also opportune.

## Ethics Statement

This study was approved by the Ethics Committee Hospital das Clinicas da Faculdade de Medicina de Ribeirão Preto—USP (CAAE: 39523314.0.0000.5440).

## Author Contributions

FO, AS, and MC—conception/design of the work; AS and CC—acquisition of data; FO, AS, CC, and MC—analysis/interpretation of data and final approval of the version to be published; and FO and MC—revising article critically for important intellectual content.

## Conflict of Interest Statement

The authors declare that the research was conducted in the absence of any commercial or financial relationships that could be construed as a potential conflict of interest.
