# Supplementary material for: Facial Recognition of Happiness Is Impaired in Musicians with High Music Performance Anxiety
Source: Front Psychiatry. 2018 Jan 25;9:5. doi: 10.3389/fpsyt.2018.00005 (PMC5810303; doi:10.3389/fpsyt.2018.00005)
Supplement: Supplementary file 1 [file Table_1.docx]

**Supplementary Material** - Illustration of the procedure Facial Expression Recognition Task Procedure - Touch the Face ( Retrieved from Donadon, 2015 – authorized)

**
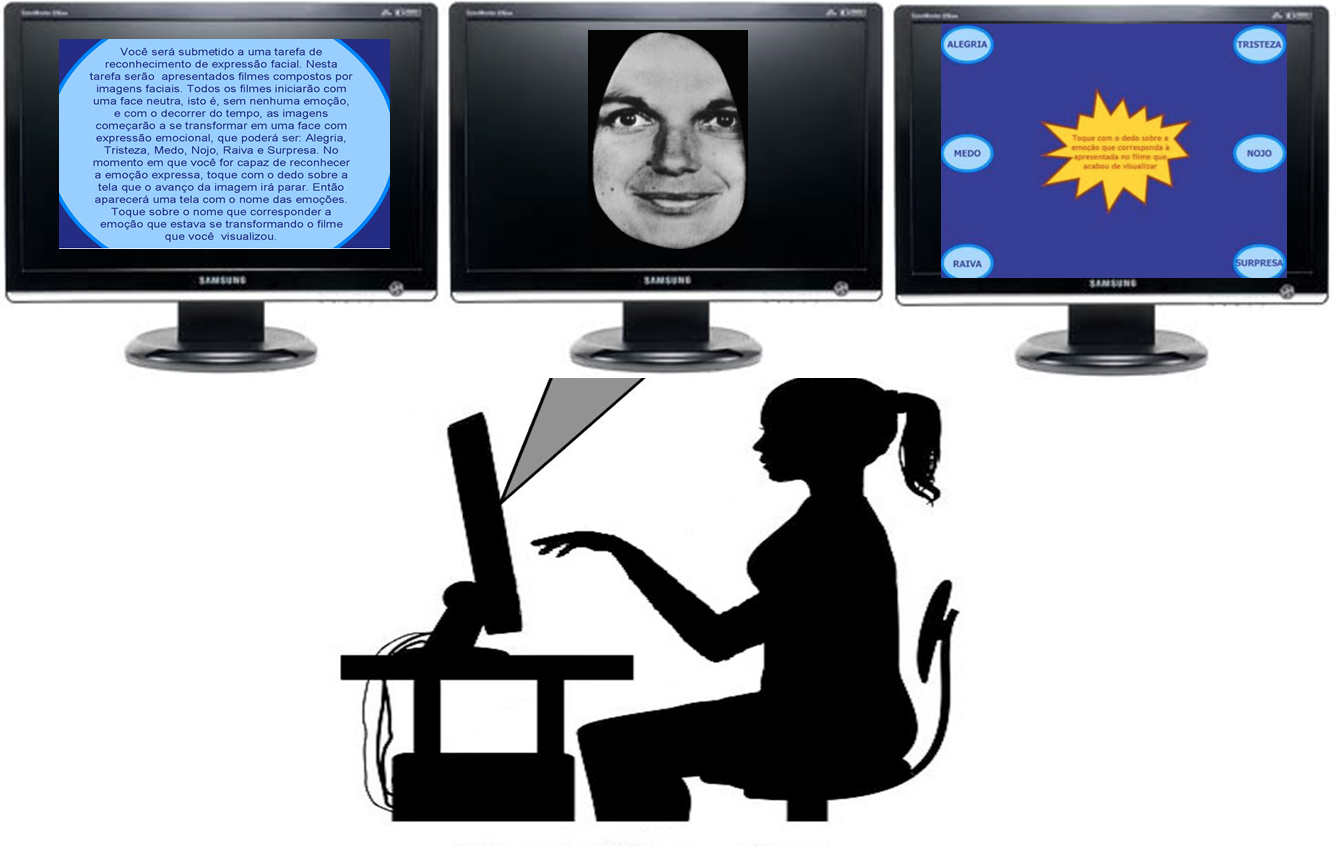
**

**Source**: Donadon MF. Alcohol addiction: associations with early emotional trauma, personality traits and recognition of facial expressions of emotion. Masters dissertation. Medical School of Ribeirão Preto. São Paulo University. São Paulo, p 119. 2015
